# Onset and progression of diabetes in kidney transplant patients receiving everolimus or cyclosporine therapy: an analysis of two randomized, multicenter trials

**DOI:** 10.1186/s12882-018-1031-1

**Published:** 2018-09-19

**Authors:** Claudia Sommerer, Oliver Witzke, Frank Lehner, Wolfgang Arns, Petra Reinke, Ute Eisenberger, Bruno Vogt, Katharina Heller, Johannes Jacobi, Markus Guba, Rolf Stahl, Ingeborg A. Hauser, Volker Kliem, Rudolf P. Wüthrich, Anja Mühlfeld, Barbara Suwelack, Michael Duerr, Eva-Maria Paulus, Martin Zeier, Martina Porstner, Klemens Budde, on behalf of the ZEUS and HERAKLES study investigators

**Affiliations:** 1 Department of Nephrology, University of Heidelberg, Im Neuenheimer Feld 162, 69120 Heidelberg, Germany; 2 Department of Infectious Diseases, University Duisburg-Essen, Essen, Germany; 3 Department of General, Visceral and Transplantation Surgery, Hannover Medical School, Hannover, Germany; 4 Department of Nephrology and Transplantation, Cologne Merheim Medical Center, Cologne, Germany; 5 Department of Nephrology and Intensive Care, Charité Campus Virchow, Charité-Universitätsmedizin Berlin, Berlin, Germany; 6 Department of Nephrology and Hypertension, University of Bern, Inselspital, Bern, Switzerland; 7 Department of Nephrology and Hypertension, University of Erlangen-Nuremberg, Erlangen, Germany; 8 Department of General-, Visceral- and Transplantation Surgery, Munich University Hospital, Campus Grosshadern, Munich, Germany; 9 Division of Nephrology, University Medical Center Hamburg-Eppendorf, Hamburg, Germany; 10 Med. Klinik III, Department of Nephrology, UKF, Goethe University, Frankfurt, Germany; 11 Department of Internal Medicine and Nephrology, Kidney Transplant Center, Nephrological Center of Lower Saxony, Klinikum Hann, Münden, Germany; 12 Division of Nephrology, University Hospital, Zürich, Switzerland; 13 Division of Nephrology and Immunology, University Hospital RWTH Aachen, Aachen, Germany; 14 Department of Internal Medicine - Transplant Nephrology, University Hospital of Münster, Münster, Germany; 15 Department of Nephrology, Charité Universitätsmedizin Berlin, Berlin, Germany; 16 Novartis Pharma GmbH, Nürnberg, Germany

**Keywords:** Diabetes, Everolimus, Kidney transplantation, TOR inhibitor, PTDM, Post-transplant

## Abstract

**Background:**

Conversion from calcineurin inhibitor (CNI) therapy to a mammalian target of rapamycin (mTOR) inhibitor following kidney transplantation may help to preserve graft function. Data are sparse, however, concerning the impact of conversion on posttransplant diabetes mellitus (PTDM) or the progression of pre-existing diabetes.

**Methods:**

PTDM and other diabetes-related parameters were assessed post hoc in two large open-label multicenter trials. Kidney transplant recipients were randomized (i) at month 4.5 to switch to everolimus or remain on a standard cyclosporine (CsA)-based regimen (ZEUS, *n* = 300), or (ii) at month 3 to switch to everolimus, remain on standard CNI therapy or convert to everolimus with reduced-exposure CsA (HERAKLES, *n* = 497).

**Results:**

There were no significant differences in the incidence of PTDM between treatment groups (log rank *p* = 0.97 [ZEUS], *p* = 0.90 [HERAKLES]). The mean change in random blood glucose from randomization to month 12 was also similar between treatment groups in both trials for patients with or without PTDM, and with or without pre-existing diabetes. The change in eGFR from randomization to month 12 showed a benefit for everolimus versus comparator groups in all subpopulations, but only reached significance in larger subgroups (no PTDM or no pre-existing diabetes).

**Conclusions:**

Within the restrictions of this post hoc analysis, including non-standardized diagnostic criteria and limited glycemia laboratory parameters, these data do not indicate any difference in the incidence or severity of PTDM with early conversion from a CsA-based regimen to everolimus, or in the progression of pre-existing diabetes.

**Trial registration:**

clinicaltrials.gov, NCT00154310 (registered September 2005) and NCT00514514 (registered August 2007); EudraCT (2006-007021-32 and 2004-004346-40).

**Electronic supplementary material:**

The online version of this article (10.1186/s12882-018-1031-1) contains supplementary material, which is available to authorized users.

## Background

Diabetic nephropathy is now the most frequent indication for kidney transplantation, accounting for approximately a third of kidney transplants in the US [1], and is set to grow in frequency as the prevalence of diabetes continues to grow [2, 3]. Additionally, under conventional immunosuppressive regimens, up to 20% of kidney transplant recipients develop posttransplant diabetes mellitus (PTDM) [4–6]. Both pre-existing diabetes [7, 8] and PTDM [9] are associated with an increased risk of cardiovascular events [9, 10] and inferior long-term survival, as well as morbidity from diabetes-related complications [7, 11]. The diabetogenic effect of calcineurin inhibitors (CNIs), particularly tacrolimus [4, 12] and steroids [5], can be compounded by maintenance steroid therapy, especially pulsed steroid therapy for the treatment of rejection [13]. In this unfavorable context, novel immunosuppressive regimens must be carefully evaluated in terms of their diabetogenic potential.

The mammalian target of rapamycin (mTOR) inhibitor agents sirolimus and everolimus have been widely assessed within a variety of regimens for de novo or delayed initiation following kidney transplantation [14]. mTOR inhibitors offer the potential for CNI sparing, which might be expected to lower the risk for PTDM [15]. However, results from the early era of sirolimus therapy in kidney transplantation raised concerns that the class may have an inherent diabetogenic effect [16, 17]. In a large randomized trial of sirolimus published in 2006, Vitko et al. reported an increased rate of PTDM in patients randomized to a loading dose (6 mg) and a fixed dose of 2 mg/day versus mycophenolate mofetil when both were administered in combination with standard-dose tacrolimus and steroids [18]. Although smaller trials using fixed sirolimus dosing [19, 20] or high sirolimus exposure targets [21] did not demonstrate any effect, an analysis of United States Renal Data System data from over 20,000 patients undergoing kidney transplantation during 1995–2003 concluded that patients treated with sirolimus were at increased risk of PTDM whether administered in combination with a CNI or an antimetabolite [22]. In contrast, a large meta-analysis published in 2006 found that use of mTOR inhibitors was not associated with any increased risk of developing insulin-treated PTDM compared to antimetabolite therapy [23].

As experience with mTOR inhibitors has grown, fixed dosing has been replaced by progressively lower trough concentration targets, and concomitant CNI exposure has been reduced [14]. Loading doses for sirolimus have typically become smaller, and no loading dose is required for everolimus due to its shorter half-life. In large, randomized trials undertaken recently, no increase in the rate of PTDM was observed in the sirolimus [24, 25] or everolimus [26, 27] treatment arms.

Conversion to an mTOR inhibitor from CNI therapy after the first 3–12 months post-transplant is an appealing immunosuppressive strategy, harnessing the potent immunosuppressive effect of CNIs during the period of highest risk for rejection but taking advantage of the reduced nephrotoxicity associated with mTOR inhibitors [28]. To date, no analyses are available concerning the impact of conversion to an mTOR inhibitor on PTDM or the progression of pre-existing diabetes. We report here a post hoc analysis of diabetic parameters in two large, multicenter trials (ZEUS [29] and HERAKLES [30]) in which de novo kidney transplant recipients were randomized to either convert to everolimus in a CNI-free regimen or to remain on a standard cyclosporine (CsA)-based regimen, or in one study to alternatively switch to everolimus with reduced-exposure CsA.

## Methods

### Study design and conduct

This was a post hoc analysis of data from two 12-month, prospective, open-label, multicenter, randomized trials of de novo kidney transplant recipients (ZEUS [29] and HERAKLES [30]).The objective of the analysis was to compare the incidence and severity of PTDM, and progression of pre-existing diabetes, to month 12 post-transplant in patients receiving everolimus-based CNI-free maintenance immunosuppression versus those who continued to receive a standard CsA-based regimen or everolimus with reduced-exposure CsA. In both studies, patients received standard-exposure CsA with enteric-coated mycophenolate sodium (EC-MPS) and steroids from time of transplant, and were randomized to continue the CsA-based regimen or convert to everolimus at 4.5 months (ZEUS) [29] or 3 months (HERAKLES) [30] post-transplant. In one of the studies (HERAKLES), there was a third treatment arm in which patients received reduced-exposure CsA with everolimus targeting a lower exposure range.

### Patients

The inclusion and exclusion criteria in the two studies were identical other than a lower maximum age for recipients and donors in the ZEUS study (65 years) versus the HERAKLES study (70 years). The minimum recipient and donor ages were 18 and 5 years, respectively, in both studies. Key exclusion criteria at time of study entry were more than one previous kidney transplant, loss of a previous graft due to immunological reasons, multiorgan transplantation, donation after cardiac death, and previous or current panel reactive antibodies > 25%. At the time of randomization, additional exclusion criteria were graft loss, severe (Banff grade ≥ III), recurrent or steroid-resistant rejection prior to randomization, proteinuria > 1 g/day and dialysis dependency. In both studies, patients with uncontrolled diabetes mellitus that in the opinion of the investigator would interfere with the appropriate conduct of the study were excluded. A full list of inclusion and exclusion criteria is shown in Additional file 1: Table S1.

### Immunosuppression

All patients received induction with basiliximab (Simulect®, Novartis Pharma, Nürnberg, Germany). CsA (Sandimmun Optoral®, Novartis Pharma, Germany) was administered to all patients, with a target trough concentration in both studies of 150–220 ng/mL from the time of transplant to randomization. All patients received EC-MPS 1440 mg/day (*myfortic®*, Novartis Pharma, Germany), and steroids administered according to local practice from the time of transplant to month 12.

For patients in the standard-CsA arms, CsA target trough concentrations after randomization were 120–180 ng/mL to month 6, and 100–150 ng/mL thereafter; EC-MPS was continued to month 12. In the CNI-free everolimus arms of both studies, everolimus was initiated at a dose of 1.5 mg then adjusted to target a trough concentration of 5–10 ng/mL, and EC-MPS was continued to month 12. In the ZEUS study, conversion from CsA to everolimus took place stepwise over a period of up to four weeks starting at month 4.5. In the HERAKLES study, conversion took place at month 3 and was completed within 24 h. In the patients randomized to everolimus with reduced-exposure CsA in the HERAKLES study, EC-MPS was discontinued and everolimus was started on the day of randomization with CsA dose unchanged, then on the following day CsA dose was adjusted to target 50–75 ng/mL thereafter. In this group, the everolimus target range was 3–8 ng/mL.

### Evaluation

This post hoc analysis compared the following outcomes between treatment groups up to month 12 post-transplant within the ZEUS and HERAKLES trials: the incidence of PTDM; requirement for hypoglycemic therapy (insulin or non-insulin); change in random blood glucose; estimated GFR (eGFR) at month 12 and the change in eGFR from randomization to month 12. Other than eGFR at month 12, none of these endpoints were pre-specified in the study protocols. Data were analyzed according to whether patients did or did not have pre-existing diabetes and subsequently did or did not develop PTDM. PTDM was defined as diabetes reported by the investigator as an adverse event at any point after transplantation in a patient not categorized as diabetic at baseline (i.e. at time of transplant). Patients were categorized as diabetic if diabetes was listed in the medical history by the investigator at the time of study entry. There were no pre-specified laboratory criteria for PTDM or pre-existing diabetes. Data on the use of insulin or other antidiabetic therapies were obtained via standard reporting procedures for concomitant medication at each study visit. If treatment with such drugs was started, investigators were required to document any adverse events, as per Good Clinical Practice guidelines. Both trials were fully monitored by an external medical monitor.

Blood glucose was measured at routine visits and are random values.

Biopsy-proven acute rejection (BPAR) was graded according to Banff criteria [31].

The primary efficacy endpoint of the ZEUS study was the adjusted eGFR estimated by the Nankivell formula (eGFR, [32]) at month 12. The primary efficacy endpoint in the HERAKLES study was change in eGFR (Nankivell formula) from randomization (month 3) to month 12.

### Statistical analysis

All analyses are reported for the safety populations, comprising all patients who received at least one dose of study drug after study entry. Data on the incidence of PTDM across both studies in patients randomized to everolimus-based CNI-free therapy or standard CsA-based therapy were pooled.

Last observation carried forward (LOCF) method was applied for missing 12-month values for immunosuppression drug doses, drug concentrations, and eGFR. Continuous variables (e.g. drug dose, drug exposure) were compared between groups using the two-sample Wilcoxon rank-sum test of the F-test. The incidence of categorical events in each study and in the pooled analysis was compared between groups using Fisher’s test. Kaplan-Meier estimates of time to events were compared between groups using the log rank test. The change in eGFR from randomization to month 12 was analyzed by an ANCOVA model with treatment, center, donor type as factors and eGFR value at randomization as covariate.

All tests were two-sided. *P* values < 0.05 were considered significant.

## Results

### Patient population and risk factors for diabetes

**Fig. 1:**
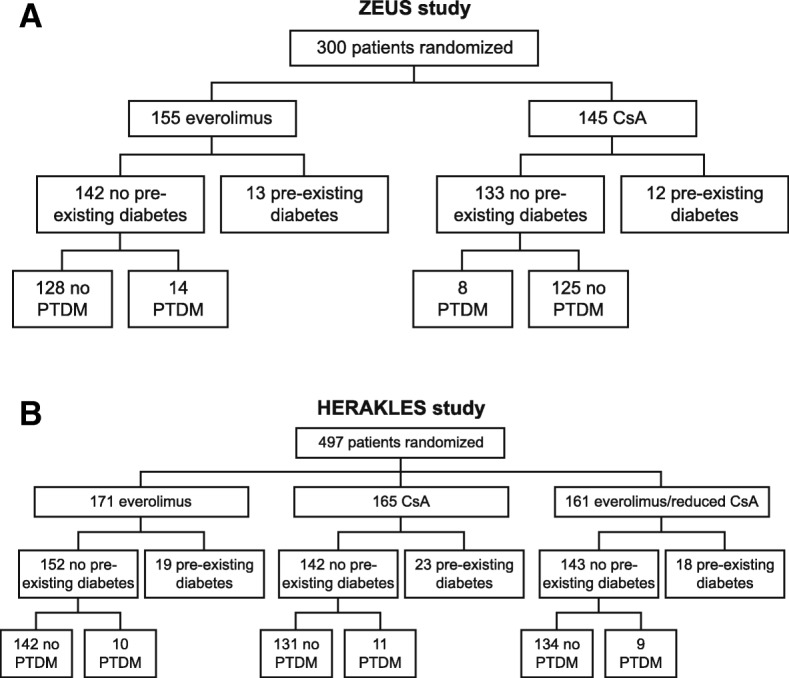
Patient disposition in (**a**) the ZEUS study (**b**) the HERAKLES study (safety populations). PTDM, posttransplant diabetes mellitus

In total, 300 patients in the ZEUS study and 497patients in the HERAKLES study were included in the current analysis. Patients were categorized according to whether they developed PTDM or whether they had pre-existing diabetes (Fig. 1).

In both studies, demographic factors, body mass index (BMI), hepatitis C (HCV) status and baseline random blood glucose concentration, generally showed no marked differences between the treatment arms among patients with PTDM, no PTDM or pre-existing diabetes (Table 1). There were significant differences between the everolimus and CsA cohorts of ZEUS for recipient age among patients with pre-existing diabetes, and for BMI at time of transplant in patients without PTDM and without pre-existing diabetes (Table 1). Almost all patients in both studies were white (97.5% [268/275] in ZEUS, 93.6% [409/437] in HERAKLES).

**Table 1:** Risk factors for diabetes in (a) the ZEUS study (b) the HERAKLES study

	PTDM			No PTDM^a^			Pre-existing diabetes			No pre-existing diabetes		
*(a) ZEUS*												
	EVR (*n* = 14)	CsA (*n* = 8)		EVR (*n* = 128)	CsA (*n* = 125)		EVR (*n* = 13)	CsA (*n* = 12)		EVR (*n* = 142)	CsA (*n* = 133)	
Recipient age at time of Tx (years)	52.9 (10.7)	57.4 (7.1)		45.8 (12.0)	45.1 (11.7)		50.7 (6.3)	56.2 (7.7)^b^		46.5 (12.0)	45.8 (11.8)	
Male gender, *n* (%)	10 (71.4)	6 (75.0)		82 (64.1)	73 (58.4)		10 (76.9)	7 (58.3)		92 (64.8)	79 (59.4)	
White recipient, *n* (%)	14 (100.0)	7 (87.5)		126 (98.4)	121 (96.8)		12 (92.3)	11 (91.7)		140 (98.6)	128 (96.2)	
BMI (kg/m^2^)												
Time of Tx	27.4 (3.0)	26.1 (3.4)		25.1 (3.9)	24.1 (3.9)^c^		28.8 (3.7)	26.9 (4.1)		25.3 (3.9)	24.2 (3.9)^d^	
Time of RDN (month 4.5)	26.9 (3.1)	28.4 (5.6)		25.7 (4.2)	25.0 (4.1)		29.1 (4.5)	27.0 (4.2)		25.8 (4.1)	25.1 (4.1)	
HCV+, n/N (%)	1/13 (7.7)	0/8 (0.0)		3/127 (2.4)	0/122 (0.0)		0/13 (0.0)	0/12 (0.0)		4/140 (2.9)	0/130 (0.0)	
Random blood glucose, mmol/L												
Time of Tx	6.3 (1.7)	6.6 (1.1)		5.4 (1.3)	5.1 (1.1)		8.9 (3.3)	9.3 (2.7)		5.5 (1.3)	5.2 (1.1)	
Time of RDN (month 4.5)	7.5 (3.9)	5.6 (1.8)		5.1 (1.0)	5.2 (1.0)		7.4 (3.2)	7.7 (3.6)		5.3 (1.3)	5.2 (1.1)	
I.V. treatment for rejection before RDN, *n* (%)	4 (28.6)	1 (12.5)		12 (9.4)	21 (16.8)		2 (15.4)	2 (16.7)		17 (12.0)	22 (16.5)	
*(b) HERAKLES*												
	EVR (*n* = 10)	CsA (*n* = 11)	EVR/reduced CsA (*n* = 9)	EVR (*n* = 142)	CsA (*n* = 131)	EVR/reduced CsA (*n* = 134)	EVR (*n* = 19)	CsA (*n* = 23)	EVR/reduced CsA (*n* = 18)	EVR (*n* = 152)	CsA (*n* = 142)	EVR/reduced CsA (*n* = 143)
Recipient age at Tx (years)	49.4 (12.3)	58.2 (6.9)	51.7 (10.9)	48.3 (12.6)	48.7 (11.9)	47.8 (12.6)	58.2 (8.1)	56.5 (9.7)	57.5 (7.1)	48.3 (12.5)	49.4 (11.8)	48.0 (12.5)
Male gender, *n* (%)	5 (50.0)	4 (36.4)	2 (22.2)	84 (59.2)	80 (61.1)	84 (62.7)	13 (68.4)	16 (69.6)	14 (77.8)	89 (58.6)	84 (59.2)	86 (60.1)
White recipient, *n* (%)	10 (100.0)	11 (100.0)	9 (100.0)	132 (93.0)	126 (96.2)	121 (90.3)	16 (84.2)	23 (100.0)	17 (94.4)	142 (93.4)	137 (96.5)	130 (90.9)
BMI (kg/m^2^)												
Time of Tx	25.0 (3.4)	28.2 (4.8)	28.2 (6.1)	24.9 (3.9)	25.3 (4.0)	25.4 (4.1)	28.8 (5.6)	30.2 (4.8)	29.4 (4.3)	24.9 (3.8)	25.5 (4.2)	25.5 (4.3)
Time of RDN (month 3)	25.3 (3.4)	28.0 (4.5)	29.3 (7.0)	25.3 (3.6)	25.4 (3.9)	25.5 (4.3)	28.7 (5.8)	29.5 (5.1)	29.2 (3.9)	25.3 (3.6)	25.6 (4.0)	25.7 (4.6)
HCV+, n/N (%)	–	–	–	1 (0.7)	3 (2.3)	2 (1.5)	–	–	–	1 (0.7)	3 (2.1)	2 (1.4)
Random blood glucose (mmol/L)												
Time of Tx	5.3 (0.9)	6.0 (1.4)	5.8 (1.1)	5.4 (1.1)	5.2 (0.8)	5.4 (1.2)	6.4 (2.7)	8.7 (3.6)	6.0 (0.9)	5.4 (1.1)	5.2 (0.9)	5.4 (1.2)
Time of RDN (month 3)	6.3 (1.7)	5.8 (0.9)	6.3 (2.4)	5.3 (1.1)	5.2 (1.1)	5.2 (1.0)	7.0 (2.4)	7.6 (3.2)	7.9 (4.1)	5.4 (1.2)	5.3 (1.1)	5.3 (1.2)
I.V. treatment for rejection before RDN, n (%)	–	1 (9.1)	2 (22.2)	14 (9.9)	7 (5.3)	12 (9.0)	4 (21.1)	4 (17.4)	1 (5.6)	14 (9.2)	8 (5.6)	14 (9.8)

^a^And no pre-existing diabetes

^b^*p* = 0.038 for everolimus versus CsA (Fisher’s test)

^c^*p* = 0.027 for everolimus versus CsA (Fisher’s test)

^d^*p* = 0.010 for everolimus versus CsA (Fisher’s test)

All differences between the everolimus and CsA groups are not significant unless stated otherwise

Continuous variables are shown as mean (SD)

*BMI* body mass index, *CsA* cyclosporine, *EVR* everolimus, *HCV+* hepatitis C virus positive, *PTDM* posttransplant diabetes mellitus, *RDN* randomization, *SD* standard deviation, *Tx* transplantation

### Immunosuppression

CsA exposure was comparable between treatment groups at randomization in both trials (Table 2). Oral steroid doses were generally slightly higher after randomization in the everolimus-based CNI-free groups than in CsA-containing regimens within the subpopulations of both trials, with the difference reaching significance in the ‘no PTDM’ and ‘no pre-existing diabetes’ groups of ZEUS (Table 2). Use of intravenous steroids to treat rejection before or after randomization was similar between treatment group arms prior to randomization in both trials, and any observed percentage differences within the PTDM and pre-existing diabetes cohorts arose from very small absolute numbers.

**Table 2:** Immunosuppression in (a) the ZEUS study and (b) the HERAKLES study

	PTDM			No PTDM^a^			Pre-existing diabetes			No pre-existing diabetes		
*(a) ZEUS*												
	EVR (*n* = 14)	CsA (*n* = 8)		EVR (*n* = 128)	CsA (*n* = 125)		EVR (*n* = 13)	CsA (*n* = 12)		EVR (*n* = 142)	CsA (*n* = 133)	
Everolimus C_0_ (ng/mL)												
RDN^b^	–	–		–	–		–	–		–	–	
M12	6.2 (1.4)	–		6.5 (2.1)	–		7.7 (2.8)	–		6.5 (2.0)	–	
CsA C_0_ (ng/mL), mean (SD)												
At RDN (month 3)	158 (25)	142 (38)		153 (51)	147 (50)		138 (63)	159 (40)		154 (49)	147 (50)	
M12	–	141 (85)		–	118 (33)		–	130 (40)		–	120 (37)	
EC-MPS dose (mg/day), mean (SD)												
RDN (month 3)	1234 (366)	1035 (449)		1317 (284)	1294 (289)		1246 (431)	1110 (419)		1309 (293)	1278 (305)	
M12	1108 (452)	1080 (465)		1193 (363)	1234 (340)		1135 (385)	1211 (333)		1186 (371)	1226 (347)	
Oral steroids(mg/day)												
BL to RDN (month 3)	21.3 (5.1)	16.3 (4.2)^c^		17.7 (5.7)	17.9 (5.7)		18.1 (6.2)	15.4 (4.8)		18.1 (5.7)	17.8 (5.6)	
RDN to M12	10.7 (7.9)	10.2 (3.2)		7.6 (2.8)	7.2 (4.3)^c^		10.3 (10.9)	6.3 (3.1)		7.9 (3.7)	7.4 (4.3)^c^	
I.V. steroids to treat rejection, *n* (%)												
BL to RDN (month 3)	4 (28.6)	1 (12.5)		13 (10.2)	21 (16.8)		2 (15.4)	2 (16.7)		17 (12.0)	22 (16.5)	
RDN to M12	3 (21.4)	1 (12.5)		12 (9.4)	11 (8.8)		1 (7.7)	2 (16.7)		15 (10.6)	12 (9.0)	
*(b) HERAKLES*												
	EVR (*n* = 10)	CsA (*n* = 11)	EVR/reduced CsA (*n* = 9)	EVR (*n* = 142)	CsA (*n* = 131)	EVR/reduced CsA (*n* = 134)	EVR (*n* = 19)	CsA (*n* = 23)	EVR/reduced CsA (*n* = 18)	EVR (*n* = 152)	CsA (*n* = 142)	EVR/reduced CsA (*n* = 143)
Everolimus C_0_ (ng/mL)												
RDN (month 4.5)	5.6 (3.0)	–	4.5 (2.1)	6.1 (3.2)	–	7.0 (12.6)	5.7 (2.1)	**–**	6.1 (2.2)	6.1 (3.1)	–	6.8 (12.2)
M12	7.2 (2.9)	–	5.7 (2.4)	6.6 (2.2)	–	6.3 (2.7)	7.2 (1.8)	**–**	5.2 (2.2)	6.6 (2.3)	–	6.3 (2.7)
CsA C_0_ (ng/mL)												
RDN (month 4.5)	155 (29)	167 (48)	157 (38)	154 (46)	164 (55)	161 (72)	169 (61)	167 (50)	157 (42)	154 (45)	164 (54)	161 (70)
M12	–	138 (56)	126 (34)	–	119 (29)	80 (34)	–	116 (36)	59 (22)	–	120 (32)	83 (35)
EC-MPS dose (mg/day)												
RDN (month 4.5)	1305 (268)	1360 (240)	1440 (0)	1345 (246)	1375 (214)	1287 (314)	1357 (216)	1271 (288)	1305 (413)	1342 (246)	1374 (215)	1296 (307)
M12	1183 (342)	1280 (317)	–	1171 (345)	1266 (335)	–	1260 (306)	1239 (336)	–	1172 (343)	1267 (332)	–
Oral steroids(mg/day)												
BL to RDN (month 4.5)	21.4 (4.4)	19.7 (5.5)^c^	20.9 (2.6)	19.2 (5.6)	19.2 (5.6)	19.3 (5.3)	16.6 (4.4)	18.4 (5.4)	18.8 (5.3)	19.4 (5.0)	19.3 (5.6)	19.4 (5.2)
RDN to M12	8.2 (3.4)	7.4 (3.2)	6.0 (2.7)	7.4 (3.6)	6.8 (3.7)	6.9 (3.1)	7.3 (4.0)	7.1 (4.2)	6.5 (1.7)	7.4(3.6)	6.9(3.6)	6.9 (3.1)
I.V. steroids to treat rejection, *n* (%)												
BL to RDN (month 4.5)	0	0	2 (22.2)	12 (8.5)	7 (5.3)	11 (8.2)	4 (21.1)	1 (4.3)	1 (5.6)	12 (7.9)	7 (4.9)	13 (9.1)
RDN to M12	1 (10.0)	2 (18.2)	1 (11.1)	13 (9.2)	10 (7.6)	12 (9.0)	4 (21.1)	2 (8.7)	1 (5.6)	14 (9.2)	12 (8.5)	13 (9.1)

^a^And no pre-existing diabetes

^b^Not recorded at RDN

^c^*p* < 0.05 for everolimus vs CsA (two-sample Wilcoxon rank-sum test of the F-test)

Continuous variables are shown as mean (SD)

Last observation carried forward (LOCF) method applied to month 12 values. All differences versus the standard CsA groups were not significant unless stated otherwise

*BL* baseline, *C*_*0*_ trough concentration, *CsA* cyclosporine, *EC-MPS* enteric-coated mycophenolate sodium, *EVR* everolimus, *I.V.* intravenous, *M12* month 12, *M6* month 6, *PTDM* posttransplant diabetes mellitus, *RDN* randomization, *SD* standard deviation

### Post-transplant diabetes mellitus

**Fig. 2:**
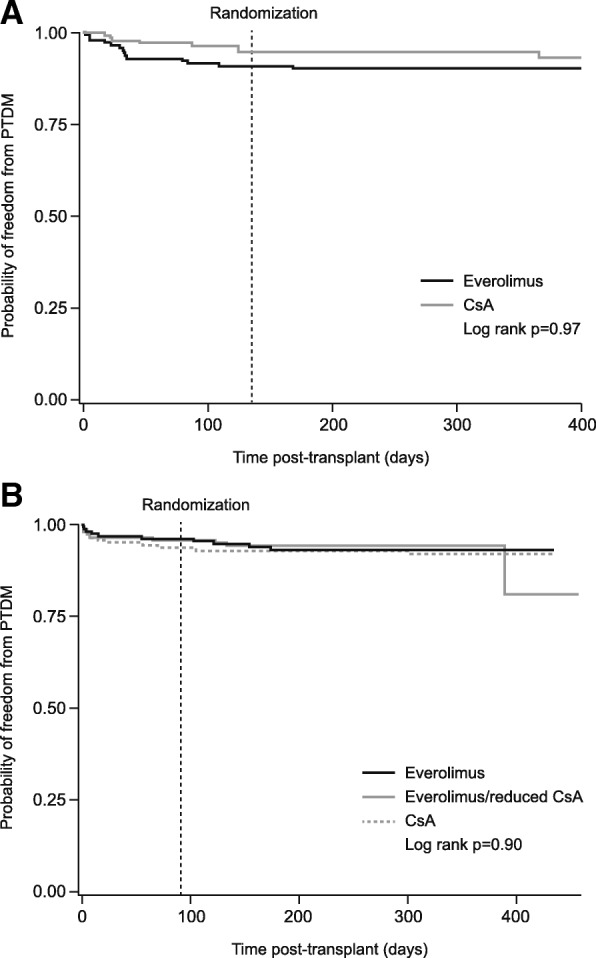
Occurrence of posttransplant diabetes mellitus (PTDM) in (**a**) the ZEUS study (**b**) the HERAKLES study (Kaplan-Meier estimates) CsA, cyclosporine

In the ZEUS study, PTDM was present at randomization (i.e. month 4.5 post-transplant) in 9.2% (13/142) everolimus-treated patients and 5.3% (7/133) of CsA-treated patients; corresponding values at month 12 were 9.9% (14/142) and 6.0% (8/133). In the HERAKLES trial, the incidence of PTDM was 4.6% (7/152), 6.3% (9/142) and 4.2% (6/143) at randomization (i.e. month 3 post-transplant) in the everolimus, CsA and everolimus/reduced CsA groups, respectively, compared to 6.6% (10/152), 7.8% (11/142) and 6.3% (9/143) at month 12. Thus, after randomization, there were only a total of two new cases of PTDM in the ZEUS study and eight new cases in the HERAKLES trial, distributed equally across treatment groups. Kaplan-Meier estimates showed that there were no significant differences in the incidence of PTDM between treatment groups in either trial (Fig. 2).

When data from both studies were pooled, the incidence of PTDM at month 12 among patients without pre-existing diabetes was 8.2% (24/294) in patients randomized to everolimus without CNI therapy, compared to 6.9% (19/275) in those randomized to standard CsA therapy (*p* = 0.64).

The use of antihyperglycemic therapy was similar between groups for patients with PTDM (everolimus 13/14 [11 insulin, 9 non-insulin therapies] patients, CsA 6/8 [6 insulin, 2 non-insulin therapies] patients) in the ZEUS study, and in the HERAKLES study (everolimus 9/10 [5 insulin, 6 non-insulin therapies], CsA 10/11 [7 insulin, 6 non-insulin therapies], everolimus/reduced CsA 9/9 [8 insulin, 6 non-insulin therapies]).

### Pre-existing diabetes

**Fig. 3:**
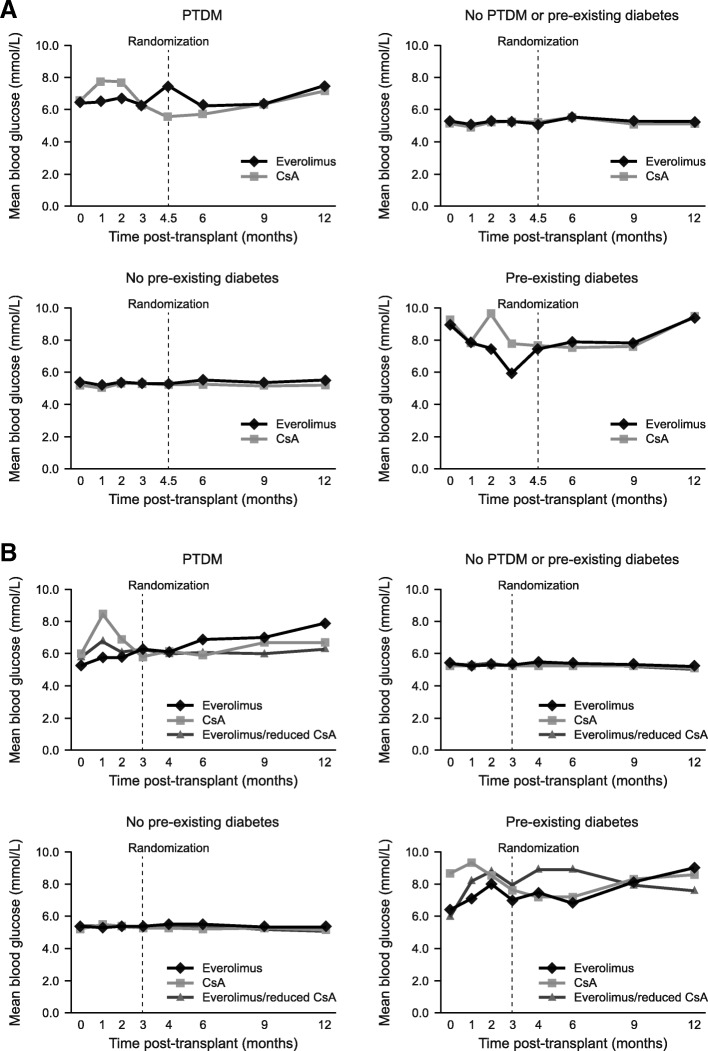
Mean random blood glucose concentrations from time of transplant to month 12 in (**a**) the ZEUS study and (**b**) the HERAKLES study. CsA, cyclosporine; PTDM, posttransplant diabetes mellitus

At time of transplant, pre-existing diabetes was present in 8.4% and 8.3% of the everolimus-treated and CsA-treated patients in the ZEUS trial, and in 11.1%, 13.9% and 11.2% of the patients randomized to everolimus, CsA or everolimus/reduced CsA in the HERAKLES study, respectively. There was no apparent difference in progression of random glucose concentrations between the two treatment groups to month 12 for patients with pre-existing diabetes (Fig. 3). From randomization to month 12, the mean (SD) change in random glucose concentration was 1.1 (3.4) mmol/L versus 1.5 (4.5)mmol/L in the everolimus versus CsA groups in the ZEUS study (*p* = 0.52). In the HERAKLES study, the mean (SD) change was 2.2 (3.0) mmol/L, 0.5 (4.9) mmol/L and − 0.2 (3.7) mmol/L in the everolimus, CsA and everolimus/reduced CsA arms, respectively (*p* = 0.24).

Among patients with pre-existing diabetes, use of antihyperglycemic therapy was similar between groups in the ZEUS trial (everolimus 12/13 patients [12 insulin, 3 non-insulin therapies], CsA 12/12 [12 insulin, 4 non-insulin therapies]) and the HERAKLES trial (everolimus 16/19 [16 insulin, 3 non-insulin], CsA 22/23 [20 insulin, 9 non-insulin], everolimus/reduced CsA17/18 [17 insulin, 6 non-insulin]).

### Blood glucose concentrations

The mean (SD) change in random blood glucose from randomization to month 12 among patients with PTDM was similar in the everolimus group versus the CsA group for the ZEUS study (*p* = 0.10) and the HERAKLES study (*p* = 0.38). Mean random blood glucose levels also remained similar between treatment groups in both trials for patients who did not develop PTDM (Fig. 3). As expected, patients with PTDM clearly had higher glucose values compared to patients without PTDM (Fig. 3).

### Biopsy-proven acute rejection

The higher rate of mild BPAR (Grade I) in the overall ZEUS study population was reflected in the cohorts without PTDM or pre-existing diabetes (Additional file 1: Table S2). In the HERAKLES study, there were no significant differences in the incidence of BPAR between treatment groups in any subpopulation (Additional file 1: Table S2).

### Renal function

The change in eGFR from randomization to month 12 was significantly in favor of everolimus in the two largest subpopulations (no PTDM and no pre-existing diabetes) for both trials (Table 3). In the subpopulations with PTDM or pre-existing diabetes, the differences between groups were of a similar order of magnitude to those seen in the larger subpopulations, but in these small cohorts statistical significance was not reached.

**Table 3:** Estimated GFR (Nankivell formula [32]) (mL/min/1.73m^2^) in (a) the ZEUS study (b) the HERAKLES study. Values are shown as mean (SD)

*(a) ZEUS study*	PTDM			No PTDM^a^			Pre-existing diabetes			No pre-existing diabetes		
	EVR (*n* = 14)		CsA (*n* = 8)	EVR (*n* = 128)		CsA (*n* = 125)	EVR (*n* = 13)	CsA (*n* = 12)		EVR (n = 142)		CsA (*n* = 133)
RDN (month 3)	64.8 (13.8)		54.3 (23.0)	63.3 (17.6)		63.0 (14.7)	67.6 (22.4)	64.4 (19.7)		63.4 (17.2)		62.5 (15.2)
P value vs CsA^b^	0.31		–	0.92		–	0.50	–		0.85		–
M12	78.5 (17.9)		49.2 (16.3)	70.4 (18.7)		61.6 (16.1)	73.1 (22.1)	65.7 (22.2)		71.1 (18.7)		61.0 (16.2)
P value vs CsA^b^	0.008		–	< 0.001			0.22			< 0.001		–
Change from RDN to M12	14.0 (11.4)		−9.2 (15.9)	7.5 (10.5)		−1.5 (9.6)	5.5 (5.9)	0.7 (10.0)		8.2 (10.8)		−1.8 (10.0)
P value vs CsA^c^	Not available^d^		–	< 0.001		–	0.87	–		< 0.001		–
*(b) HERAKLES study*	PTDM			No PTDM^a^			Pre-existing diabetes			No pre-existing diabetes		
	EVR (*n* = 10)	CsA (*n* = 11)	EVR/reduced CsA (*n* = 9)	EVR (*n* = 142)	CsA (*n* = 131)	EVR/reduced CsA (*n* = 134)	EVR (*n* = 19)	CsA (*n* = 23)	EVR/reduced CsA (*n* = 18)	EVR (*n* = 152)	CsA (*n* = 142)	EVR/reduced CsA (*n* = 143)
RND (month 4.5)	70.6 (22.1)	62.4 (16.7)	59.4 (23.4)	64.9 (14.6)	61.2 (15.1)	62.8 (14.1)	71.3 (17.9)	65.1 (13.6)	67.6 (15.2)	65.3 (15.2)	61.3 (15.2)	62.6 (14.8)
P value vs CsA^b^	0.34	–	0.81	0.05	–	0.39	0.41	–	0.59	0.03	–	0.43
M12	75.5 (29.9)	60.5 (26.3)	56.4 (28.1)	70.8 (18.3)	62.2 (16.1)	63.1 (18.9)	76.5 (20.8)	65.5 (16.8)	70.5 (19.8)	71.1 (19.2)	62.1 (17.0)	62.7 (19.5)
P value vs CsA^b^	0.19	–	1.0	< 0.001	–	0.61	0.11	–	0.50	< 0.001	–	0.63
Change from RDN to M12	4.9 (16.7)	−1.9 (15.8)	−3.0 (10.1)	5.9 (13.9)	1.0 (11.1)	0.4 (12.8)	5.3 (12.9)	2.8 (11.8)	0.1 (11.7)	5.8 (14.0)	0.7 (11.5)	0.1 (12.7)
P value vs CsA^c^	0.11	–	0.86	< 0.001	–	< 0.001	0.17	–	0.78	< 0.001	–	< 0.001

^a^And no pre-existing diabetes

^b^*P* values based on the two-sample Wilcoxon rank-sum test of the F-test

^c^*P* values based on ANCOVA model with treatment, center, donor type as factors and estimated GFR value at randomization as covariate

^d^Numbers too low to permit a meaningful ANCOVA analysis

Last observation carried forward (LOCF) method applied to month 12 values

*CsA* cyclosporine, *EVR* everolimus, *GFR* glomerular filtration rate, *M12* month 12, *PTDM* posttransplant diabetes mellitus, *RDN* randomization

## Discussion

Results from this post hoc analysis of two large randomized studies do not suggest any difference in the incidence or severity of PTDM in patients who were converted early post-transplant from a CsA-based regimen to everolimus, or in the progression of pre-existing diabetes. Kaplan-Meier estimates showed comparable rates of PTDM in the different treatment cohorts after randomization in both studies to month 12, with similar patterns of random blood glucose concentration over time in the subpopulations with or without PTDM or pre-existing diabetes. The number of patients who developed PTDM after randomization was identical in the everolimus group or the standard CsA group, but absolute numbers were very low, even within this large pooled cohort, so firm conclusions cannot be drawn.

The progressive renal deterioration which is frequently associated with diabetes in the general population has also been documented in patients with PTDM [33, 34], so any potential benefit for preservation of renal function may be particularly relevant in this subpopulation. Here, the change in eGFR from randomization to month 12 in patients receiving everolimus within a CNI-free regimen which was achieved in the overall study populations at month 12 [29, 30] was also observed in the subpopulation of patients with PTDM, although the small numbers of patients precluded any statistical differences. Mean eGFR in patients randomized to CNI-free therapy with everolimus in patients with PTDM improved by 14 mL/min/1.73m^2^ in the ZEUS study and by 4.9 mL/min/1.73m^2^ in HERAKLES by month 12. Among patients with pre-existing diabetes, there was a numerically greater improvement in renal function from randomization to month 12 in the CNI-free cohorts of both trials versus the comparator groups.

Certain aspects of the analysis should be taken into account. First, PTDM was included from the time of transplant, with few events in either group after randomization, and the onset of PTDM was collected by standard adverse event reporting, with no pre-specified criteria. In the DIRECT study [4], in which data on HbA1_c_, insulin level and oral glucose tolerance testing were collected prospectively to month 6 in patients receiving a standard CsA-based regimen, the incidence of treated PTDM was 7.4% but a further 7.4% had untreated PTDM based on American Diabetes Association criteria (14.8% overall) [35]. Based on the 6.8–8.0% incidence of PTDM reported by 12 months in the current studies, it is likely that the standard reporting adverse event techniques did not capture all cases of PTDM. However, since it seems reasonable to assume that centers applied the same procedures for monitoring and defining PTDM regardless of patients’ immunosuppressive treatment, it is unlikely that this will have biased diagnosis rates between treatment arms. Second, oral glucose tolerance tests were not recorded and HbA1_c_ levels were not measured, with the only available laboratory test being random blood glucose levels. This is a weakness of the analysis. The presence of hyperglycemia could be recorded as an adverse event by investigators, but no strict reporting checks for this value were in place, hence reporting of hyperglycemia is not fully reliable. Information on antidiabetic therapy was captured by standard documentation of concomitant medication, and may have been incomplete despite the strict external monitoring. Third, the analysis does not address the relative diabetogenic effect of everolimus and CsA during the very early post-transplant period (up to month 3), when the rate of onset of glucose metabolism disturbances, hyperglycemia and onset of PTDM can be highest [4, 36]. Fourth, the results presented here apply only to conversion from CsA (not tacrolimus) to everolimus after the initial weeks post-transplant. Tacrolimus, now the dominant CNI, is widely regarded to be more diabetogenic than CsA [37, 38]. Fifth, the studies were not powered to detect differences within subpopulations for the primary endpoint (eGFR at month 12) or to detect differences in any of the endpoints which were specified post hoc. Indeed, even with this large pooled dataset, the number of patients who developed PTDM after randomization was very small, restricting interpretation. Lastly, the follow-up period (a maximum of nine months after randomization) was too short to assess any long-term effect of CNI administration on late-onset PTDM or progression of pre-existing diabetes.

## Conclusions

Within the limitations of this post hoc analysis, including an absence of pre-specified diagnostic criteria for PTDM or extensive laboratory data, conversion from a CsA-based regimen to everolimus in combination with mycophenolic acid and steroids within the first six months after kidney transplantation does not appear to affect the subsequent risk of developing PTDM, or adversely affect the progression of pre-existing diabetes. This finding from the subgroup analysis adds to the observed benefit on renal function after switching to everolimus-based therapy with CNI-withdrawal seen from the main study analyses.

## Additional file

Additional file 1: **Table S1.** Inclusion and exclusion criteria. **Table S2.** Efficacy endpoints between randomization and month 12 post-transplant in (a) the ZEUS study (b) the HERAKLES study. (DOCX 24 kb)

## Abbreviations

BMI: Body mass index
BPAR: Biopsy-proven acute rejection
CNI: Calcineurin inhibitor
CsA: Cyclosporine
EC-MPS: Enteric-coated mycophenolate sodium
eGFR: Estimated glomerular filtration rate
HCV: Hepatitis C virus
LOCF: Last observation carried forward
mTOR: Mammalian target of rapamycin
PTDM: Posttransplant diabetes mellitus
SD: Standard deviation

## References

[1] https://optn.transplant.hrsa.gov/data/about-data/optn-database/ (Organ Procurement and Transplantation Network [OPTN] National Data Reports, Waiting list, Organ by diagnosis) Accessed 13 Mar 2016.

[2] Adeghate E, Schattner P, Dunn E (2006). An update on the etiology and epidemiology of diabetes mellitus. Ann N Y Acad Sci.

[3] Herman WH, Zimmet P (2012). Type 2 diabetes: an epidemic requiring global attention and urgent action. Diabetes Care.

[4] Vincenti F., Friman S., Scheuermann E., Rostaing L., Jenssen T., Campistol J. M., Uchida K., Pescovitz M. D., Marchetti P., Tuncer M., Citterio F., Wiecek A., Chadban S., El-Shahawy M., Budde K., Goto N. (2007). Results of an International, Randomized Trial Comparing Glucose Metabolism Disorders and Outcome with Cyclosporine Versus Tacrolimus. American Journal of Transplantation.

[5] Chadban SJ (2008). New-onset diabetes after transplantation--should it be a factor in choosing an immunosuppressant regimen for kidney transplant recipients. Nephrol Dial Transplant.

[6] Sarno G, Muscogiuri G, De Rosa P (2012). New-onset diabetes after kidney transplantation: prevalence, risk factors, and management. Transplantation.

[7] Boucek P, Saudek F, Pokorna E (2002). Kidney transplantation in type 2 diabetic patients: a comparison with matched non-diabetic subjects. Nephrol Dial Transplant.

[8] Rømming Sørensen V, Schwartz Sørensen S, Feldt-Rasmussen B (2006). Long-term graft and patient survival following renal transplantation in diabetic patients. Scand J Urol Nephrol.

[9] Hjelmesaeth J, Hartmann A, Leivestad T (2006). The impact of early-diagnosed new-onset post-transplantation diabetes mellitus on survival and major cardiac events. Kidney Int.

[10] Cosio FG, Kudva Y, van der Velde M (2005). New onset hyperglycemia and diabetes are associated with increased cardiovascular risk after kidney transplantation. Kidney Int.

[11] Burroughs TE, Swindle J, Takemoto S (2007). Diabetic complications associated with new-onset diabetes mellitus in renal transplant recipients. Transplantation.

[12] Heisel O, Heisel R, Balshaw R, Keown P (2004). New onset diabetes mellitus in patients receiving calcineurin inhibitors: a systematic review and meta-analysis. Am J Transplant.

[13] Vesco L, Busson M, Bedrossian J, Bitker MO, Hiesse C, Lang P (1996). Diabetes mellitus after renal transplantation: characteristics, outcome, and risk factors. Transplantation.

[14] Weir MR, Diekmann F, Flechner SM (2010). mTOR inhibition: the learning curve in kidney transplantation. Transpl Int.

[15] Sharif A, Shabir S, Chand S, Cockwell P, Ball S, Borrows R (2011). Meta-analysis of calcineurin-inhibitor-sparing regimens in kidney transplantation. J Am Soc Nephrol.

[16] Sharif A, Hecking M, de Vries AP (2014). Proceedings from an international consensus meeting on posttransplantation diabetes mellitus: recommendations and future directions. Am J Transplant.

[17] Liefeldt L, Budde K (2010). Risk factors for cardiovascular disease in renal transplant recipients and strategies to minimize risk. Transpl Int.

[18] Vitko S, Wlodarczyk Z, Kyllönen L (2006). Tacrolimus combined with two different dosages of sirolimus in kidney transplantation: results of a multicenter study. Am J Transplant.

[19] Sampaio EL, Pinheiro-Machado PG, Garcia R, et al. Mycophenolate mofetil vs. sirolimus in kidney transplant recipients receiving tacrolimus-based immunosuppressive regimen. Clin Transpl. 2008;22(2):141–149. 10.1111/j.1399-0012.2007.00756.x 18339132

[20] Machado PG, Felipe CR, Hanzawa NM (2004). An open-label randomized trial of the safety and efficacy of sirolimus vs. azathioprine in living related renal allograft recipients receiving cyclosporine and prednisone combination. Clin Transpl.

[21] Groth Carl G., Bäckman Lars, Morales José-Maria, Calne Roy, Kreis Henri, Lang Philippe, Touraine Jean-Louis, Claesson Kerstin, Campistol Josep M., Durand Dominique, Wramner Lars, Brattström Christina, Charpentier Bernard (1999). SIROLIMUS (RAPAMYCIN)-BASED THERAPY IN HUMAN RENAL TRANSPLANTATION. Transplantation.

[22] Johnston W, Rose CL, Webster AC, Gill JS (2008). Sirolimus is associated with new-onset diabetes in kidney transplant recipients. J Am Soc Nephrol.

[23] Webster AC, Lee VWS, Chapman JR, Craig JC (2006). Target of rapamycin inhibitors (sirolimus and everolimus) for primary immunosuppression of kidney transplant recipients: a systematic review and meta-analysis of randomized trials. Transplantation.

[24] Flechner SM, Glyda M, Cockfield S (2011). The ORION study: comparison of two sirolimus-based regimens versus tacrolimus and mycophenolate mofetil in renal allograft recipients. Am J Transplant.

[25] Ekberg H, Tedesco-Silva H, Demirbas A (2007). ELITE-Symphony Study. Reduced exposure to calcineurin inhibitors in renal transplantation. N Engl J Med.

[26] Tedesco Silva H, Cibrik D, Johnston T (2010). Everolimus plus reduced-exposure CsA versus mycophenolic acid plus standard-exposure CsA in renal-transplant recipients. Am J Transplant.

[27] Qazi Y, Shaffer D, Kaplan B (2017). Efficacy and safety of everolimus plus low-dose tacrolimus versus mycophenolate mofetil plus standard-dose tacrolimus in de novo renal transplant recipients: 12-month data. Am J Transplant.

[28] Rostaing L, Kamar N (2010). mTOR inhibitor/proliferation signal inhibitors: entering or leaving the field?. J Nephrol.

[29] Budde Klemens, Becker Thomas, Arns Wolfgang, Sommerer Claudia, Reinke Petra, Eisenberger Ute, Kramer Stefan, Fischer Wolfgang, Gschaidmeier Harald, Pietruck Frank (2011). Everolimus-based, calcineurin-inhibitor-free regimen in recipients of de-novo kidney transplants: an open-label, randomised, controlled trial. The Lancet.

[30] Budde K, Zeier M, Witzke O (2017). Everolimus with cyclosporine withdrawal or low-exposure cyclosporine in kidney transplantation from month 3: a multicentre, randomized trial. Nephrol Dial Transplant.

[31] Racusen LC, Solez K, Colvin RB (1999). The Banff 97 working classification of renal allograft pathology. Kidney Int.

[32] Nankivell BJ, Gruenewald SM, Allen R, Chapman JR (1995). Predicting glomerular filtration rate after kidney transplantation. Transplantation.

[33] Madhav D, Ram R, Dakshinamurty KV (2010). Posttransplant diabetes mellitus: analysis of risk factors, effects on biochemical parameters and graft function 5 years after renal transplantation. Transplant Proc.

[34] Pietrzak-Nowacka M, Safranow K, Dziewanowski K (2008). Impact of posttransplant diabetes mellitus on graft function in autosomal dominant polycystic kidney disease patients after kidney transplantation. Ann Acad Med Stetin.

[35] American Diabetes Association (2003). Expert Committee on the Diagnosis and Classification of Diabetes Mellitus.Report of the expert committee on the diagnosis and classification of diabetes mellitus. Diabetes Care.

[36] Luan FL, Stuckey LJ, Ojo AO (2010). Abnormal glucose metabolism and metabolic syndrome in non-diabetic kidney transplant recipients early after transplantation. Transplantation.

[37] Wissing Karl M., Abramowicz Daniel, Weekers Laurent, Budde Klemens, Rath Thomas, Witzke Oliver, Broeders Nilufer, Kianda Mireille, Kuypers Dirk R. J. (2018). Prospective randomized study of conversion from tacrolimus to cyclosporine A to improve glucose metabolism in patients with posttransplant diabetes mellitus after renal transplantation. American Journal of Transplantation.

[38] Knoll GA, Bell RC (1999). Tacrolimus versus cyclosporin for immunosuppression in renal transplantation: meta-analysis of randomised trials. BMJ.

