# Supplementary material for: Onset and progression of diabetes in kidney transplant patients receiving everolimus or cyclosporine therapy: an analysis of two randomized, multicenter trials
Source: BMC Nephrol. 2018 Sep 19;19:237. doi: 10.1186/s12882-018-1031-1 (PMC6146542; doi:10.1186/s12882-018-1031-1)
Supplement: Supplementary file 1 — Table S1. Inclusion and exclusion criteria. Table S2. Efficacy endpoints between randomization and month 12 post-transplant in (a) the ZEUS study (b) the HERAKLES study. (DOCX 24 kb) [file 12882_2018_1031_MOESM1_ESM.docx]

**Table S1** Inclusion and exclusion criteria.

| **ZEUS Study** |
| --- |
| ***Criteria at screening visit (prior to transplantation)*** |
| *Inclusion criteria*   1. Males or females, aged 18–65 years 2. Recipients of de novo cadaveric, living unrelated or living related kidney transplants 3. Females capable of becoming pregnant must have a negative serum pregnancy test within 7 days prior to or at screening, and are required to practice an approved method of birth control for the duration of the study and for a period of 6 weeks following discontinuation of study medication, even where there has been a history of infertility. 4. Patients who are willing and able to participate in the study and from whom written informed consent has been obtained. |
| *Exclusion criteria*   1. More than one previous renal transplantation 2. Multi-organ recipients (e.g., kidney and pancreas) or previous transplant with any non-renal organ 3. Patients receiving a kidney from a non-heart beating donor 4. Donor age: <5 years and >65 years 5. Graft loss due to immunological reasons in the first year after transplantation (in case of secondary transplantation) 6. Patients who are recipients of ABO incompatible transplants 7. Patients with a historical or current peak PRA of >25% 8. Patients with already existing antibodies against the HLA-type of the receiving transplant 9. Patients with any known hypersensitivity to basiliximab, everolimus, mycophenolic acid, CsA, other drugs similar to everolimus (e.g., macrolides), or other components of the formulations (e.g. lactose) 10. Patients who have received an investigational immunosuppressive drug within four weeks prior to study entry 11. Patients with thrombocytopenia (platelets <75,000/mm³), with an absolute neutrophil count of  <1,500/mm³ or leucopenia (leucocytes <2,500/mm³), or hemoglobin <6 g/dL 12. Patients with symptoms of significant somatic or mental illness. Inability to cooperate or communicate with the investigator, who are unlikely to comply with the study requirements, or who are unable to give informed consent 13. Patients with a history of malignancy during the last five years, except squamous or basal cell carcinoma of the skin 14. Patients who are HIV positive or hepatitis B surface antigen positive or hepatitis C virus positive. Recipients of organs from donors who test positive for hepatitis B surface antigen or hepatitis C are excluded. 15. Evidence of severe liver disease (including abnormal liver enzyme profile, i.e. AST, ALT or total bilirubin >3 times ULN) 16. Females of childbearing potential who are planning to become pregnant, who are pregnant or lactating, and/or who are unwilling to use effective means of contraception 17. Presence of a clinically significant infection requiring continued therapy, severe diarrhea, active peptic ulcer disease, or uncontrolled diabetes mellitus that in the opinion of the investigator would interfere with the appropriate conduct of the study 18. Evidence of drug or alcohol abuse 19. Patients receiving drugs known to interact with CsA and/or everolimus. |
| ***Criteria at randomization*** |
| *Inclusion criteria*   1. Patients have to be on an immunosuppressive regimen with enteric-coated mycophenolic sodium (target dose: 1440 mg/day, if tolerated; minimal dose: 720 mg/day), CsA, and corticosteroids. 2. Patients with serum creatinine ≤3.0 mg/dL |
| *Exclusion criteria*   1. Graft loss 2. Changes to the immunosuppressive regimen prior to randomization due to immunologic reasons. 3. Patients who suffered from severe rejection (more than or equal to BANFF II acute rejection), recurrent acute rejection, or steroid resistant acute rejection. 4. Patients with thrombocytopenia (platelets < 75,000/mm³), with an absolute neutrophil count of <1,500/mm³ or leucopenia (leucocytes <2,500/mm³), or hemoglobin <6 g/dL 5. Evidence of severe liver disease (incl. abnormal liver enzyme profile, i.e. AST, ALT or total bilirubin >3 times ULN) 6. Proteinuria >1g/day 7. Dialysis dependency at randomization visit. 8. Patients with clinically significant infection requiring continued therapy which would interfere with the objectives of the study 9. Presence of intractable immunosuppressant complications or side effects (e.g., severe gastrointestinal adverse events) at randomization visit |
| **HERAKLES study** |
| ***Criteria at screening visit (prior to transplantation)*** |
| *Inclusion criteria*  1. Males or females, aged 18–70 years  2. Recipients of de novo cadaveric, living unrelated or living related kidney transplants  3. Females capable of becoming pregnant must have a negative serum pregnancy test within 7 days  prior to or at the screening visit, and are required to practice an approved method of birth control for  the duration of the study and for a period of 6 weeks following discontinuation of study medication,  even where there has been a history of infertility.  4. Patients who are willing and able to participate in the study and from whom written informed  consent has been obtained. |
| *Exclusion criteria* 1. More than one previous renal transplantation  2. Multi-organ recipients (e.g., kidney and pancreas) or previous transplant with any other non-renal organ  3. Patients receiving a kidney from a non-heart beating donor  4. Donor age: <5 years or >70 years  5. Graft loss due to immunological reasons in the first year after transplantation (in case of  secondary transplantation)  6. Patients who are recipients of ABO incompatible transplants  7. Patients with a historical or current (≤3 months) peak PRA of >25%  8. Patients with already existing antibodies against the HLA-type of the receiving transplant  9. Patients with any known hypersensitivity to basiliximab, everolimus, mycophenolic acid, CsA  A, other drugs similar to everolimus (e.g., macrolides), or other components of the formulations (e.g.  lactose)  10. Patients who have received an investigational immunosuppressive drug within four weeks prior to  study entry  11. Patients with thrombocytopenia (platelets <75,000/mm³), with an absolute neutrophil count of  <1,500/mm³ or leucopenia (leucocytes 2,500/mm³), or hemoglobin <6 g/dL  12. Patients with symptoms of significant somatic or mental illness. Inability to cooperate or  communicate with the investigator, who are unlikely to comply with the study requirements, or who  are unable to give informed consent  13. Patients with a history of malignancy during the last five years, except squamous or basal cell  carcinoma of the skin  14. Patients who are HIV, HCV, or hepatitis B surface antigen positive  15. Evidence of severe liver disease (including abnormal liver enzyme profile, i.e. AST, ALT or total  bilirubin >3 times ULN)  16. Females of childbearing potential who are planning to become pregnant, who are pregnant or  lactating, and/or who are unwilling to use effective means of contraception  17. Presence of a clinically significant infection requiring continued therapy, severe diarrhea, active  peptic ulcer disease, or uncontrolled diabetes mellitus that in the opinion of the investigator would  interfere with the appropriate conduct of the study  18. Evidence of drug or alcohol abuse  19. Patients receiving drugs known to strongly interact with CsA and/or everolimus should be excluded, if in the opinion of the investigator this drug interaction interferes with the objectives of the study, namely a clinical meaningful potentiation of renal dysfunction and/or maintenance of adequate immunosuppressive drug levels |
| ***Criteria at randomization*** |
| *Inclusion criteria*  1. Patients have to be on an immunosuppressive regimen with enteric-coated mycophenolate sodium (target dose: 1440 mg/day, if tolerated; minimal dose: 720 mg/day), CsA, and corticosteroids.  2. Patients with serum creatinine ≤3.0 mg/dL |
| *Exclusion criteria*  1. Graft loss after current transplantation  2. Changes to the immunosuppressive regimen prior to randomization due to immunologic reasons.  3. Patients who suffered from severe rejection (more than or equal to BANFF II acute rejection),  recurrent acute rejection, or steroid resistant acute rejection.  4. Patients with thrombocytopenia (platelets <75,000/mm³), with an absolute neutrophil count of <1,500/mm³ or leucopenia (leucocytes <2,500/mm³), or hemoglobin <6 g/dL  5. Evidence of severe liver disease (including abnormal liver enzyme profile, i.e. AST, ALT or total  bilirubin >3 times ULN)  6. Proteinuria >1g/day  7. Current dialysis dependency  8. Patients with clinically significant infection requiring continued therapy which would interfere with  the objectives of the study  9. Presence of intractable immunosuppressant complications or side effects (e.g., severe  gastrointestinal adverse events) at randomization visit |

**Table S2** Efficacy endpoints between randomization and month 12 in (a) the ZEUS study (b) the HERAKLES study

|  | **PTDM** | | **No PTDM^a^** | | **Pre-existing diabetes** | | **No pre-existing diabetes** | |
| --- | --- | --- | --- | --- | --- | --- | --- | --- |
|  | **Everolimus (n=14)** | **CsA (n=8)** | **Everolimus (n=128)** | **CsA (n=125)** | **Everolimus (n=13)** | **CsA (n=12)** | **Everolimus (n=142)** | **CsA (n=133)** |
| *(a) ZEUS* | | | | | | | | |
| BPAR  Grade I  Grade II | 2 (14.3) 2 (14.3) 0 | 1 (12.5) 1 (12.5)  0 | 12 (9.4) 11 (8.6) 1 (0.8) | 4 (3.2) 3 (2.4)^b^ 1 (0.8) | 1 (7.7) 1 (7.7) 0 | 0 0 0 | 14 (9.9) 13 (9.2) 1 (0.7) | 5 (3.8) 4 (3.0)^c^ 1 (0.8) |
| Graft loss | 0 | 0 | 0 | 0 | 0 | 0 | 0 | 0 |
| Death | 0 | 1 (12.5) | 0 | 0 | 0 | 0 | 0 | 1 (0.8) |

| *(b) HERAKLES* | **PTDM** | | | **No PTDM^a^** | | | **Pre-existing diabetes** | | | **No pre-existing diabetes** | | |
| --- | --- | --- | --- | --- | --- | --- | --- | --- | --- | --- | --- | --- |
|  | **EVR (n=10)** | **CsA  (n=11)** | **EVR/ reduced CsA (n=9)** | **EVR (n=142)** | **CsA  (n=131)** | **EVR/ reduced CsA (n=134)** | **EVR (n=19)** | **CsA  (n=23)** | **EVR/ reduced CsA (n=18)** | **EVR (n=152)** | **CsA  (n=142)** | **EVR/ reduced CsA (n=143)** |
| BPAR  Grade I  Grade II  Missing | 1 (10.0) 1 (10.0) 0 | 2 (18.2) 1 (9.1) 1 (9.1) | 1 (11.1) 0 1 (11.1) | 17(12.0) 13 (9.2) 1 (0.7) 3 (2.3) | 10 (7.6) 6 (4.6) 2 (2.3) 1 (0.8) | 12 (9.0) 8 (6.0)  2 (1.5) 2 (1.5) | 2 (10.5) 2 (10.5) 0 | 1 (4.3) 1 (4.3) 0 | 0 0 0 | 18(11.8) 14 (9.2) 1 (0.7) 3 (2.0) | 12 (8.5) 7 (4.9) 4 (2.8) 1 (0.7) | 13 (9.1) 8 (5.6) 3 (2.1) 2 (1.4) |
| Graft loss | 0 | 1 (9.1) | 1 (11.1) | 1 (0.7) | 0 | 0 | 0 | 0 | 0 | 1 (0.7) | 1 (0.7) | 1 (0.7) |
| Death | 0 | 0 | 0 | 1 (0.7) | 2 (1.6) | 2 (1.5) | 0 | 0 | 0 | 1 (0.7) | 2 (1.4) | 2 (1.4) |

^a^ And no pre-existing diabetes

^b^ p=0.051
^c^ p=0.044

P values are shown versus the standard CsA group for each study. P values based on Fishers Exact Test

All differences between the treatment groups are not significant unless stated otherwise

BPAR, biopsy-proven acute rejection; CsA, cyclosporine; EVR, everolimus; PTDM, posttransplant diabetes mellitus
